# Ectopic cerebellar tissue in the occipital bone: a case report

**DOI:** 10.1186/s13256-017-1394-0

**Published:** 2017-08-21

**Authors:** Mariko Kawashima, Masahito Kobayashi, Keisuke Ishizawa, Takamitsu Fujimaki

**Affiliations:** 1 Department of Neurosurgery, Saitama Medical University Hospital, 38 Morohongo, Moroyama-machi, Iruma, Saitama 350-0495 Japan; 2 Department of Pathology, Saitama Medical University Hospital, Saitama, Japan

**Keywords:** Ectopic cerebellum, Glioneuronal ectopia, Intraosseous lesion, Occipital bone

## Abstract

**Background:**

Ectopic cerebellar tissue located distantly from the normal cerebellum is very rare, and its pathophysiology remains to be elucidated.

**Case presentation:**

We report an extremely rare case of intraosseous ectopic cerebellum detected incidentally at suboccipital craniotomy in a 46-year-old Japanese woman with hemifacial spasm. She had a small bone defect in the occipital bone, which contained a tiny area of soft tissue surrounded by cerebrospinal fluid connecting to the normal subarachnoid space through a dural opening. Histopathology demonstrated cerebellar cortex tissue consisting of molecular and granular cell layers.

**Conclusions:**

This is the first report of glioneuronal ectopia within the skull bone separated from normal brain tissue, and it is important to distinguish this entity from other osteolytic lesions.

## Background

Ectopic cerebellar tissue located distantly from the normal cerebellum is very rare, with 13 previously reported cases in the literature, of which only one was an adult case. This is obviously different from tonsillar herniation with Chiari malformations and its pathogenesis or association with any syndrome remains to be elucidated. This report describes the first known case of ectopic cerebellar tissue in the skull and discusses the differential diagnosis and pathogenesis of ectopic cerebellum.

## Case presentation

**Fig. 1:**
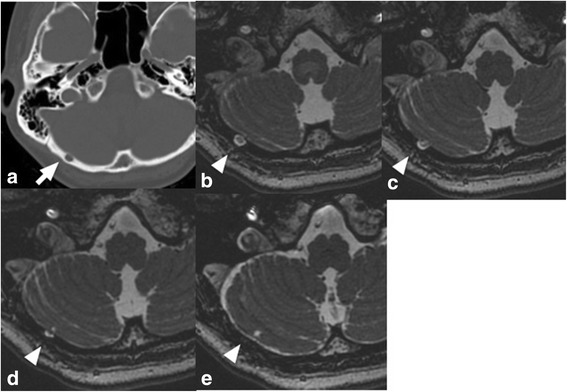
Non-contrast computed tomography via a bone window reveals an oval bone defect in the right occipital bone (**a**, *arrow*), and features shown by magnetic resonance imaging using constructive interference in steady state axial sequences (**b**, **c**, **d**, **e**, *arrowheads*)

A 46-year-old Japanese woman presented with right hemifacial spasm that had persisted for 2 years, and was admitted to our hospital for microvascular decompression. She had shown no medical abnormality during the perinatal period and had developed normally. Furthermore, she had no history of malignant diseases or severe head injury, or any remarkable family history. Findings of physical and neurological examinations were unremarkable except for right hemifacial spasm with synkinesis. A head computed tomography (CT) scan revealed a small intraosseous defect 6 mm in diameter in the right occipital bone. Magnetic resonance (MR) images obtained using heavily T2-weighted MR cisternography showed an isointense mass 6 mm in diameter outside the right cerebellar hemisphere, corresponding to the bone defect. Around the mass, there was a high-intensity area similar to cerebrospinal fluid (CSF), which was continuous with the intracranial subarachnoid space (Fig. 1), indicating that the mass was located within the subarachnoid space.

**Fig. 2:**
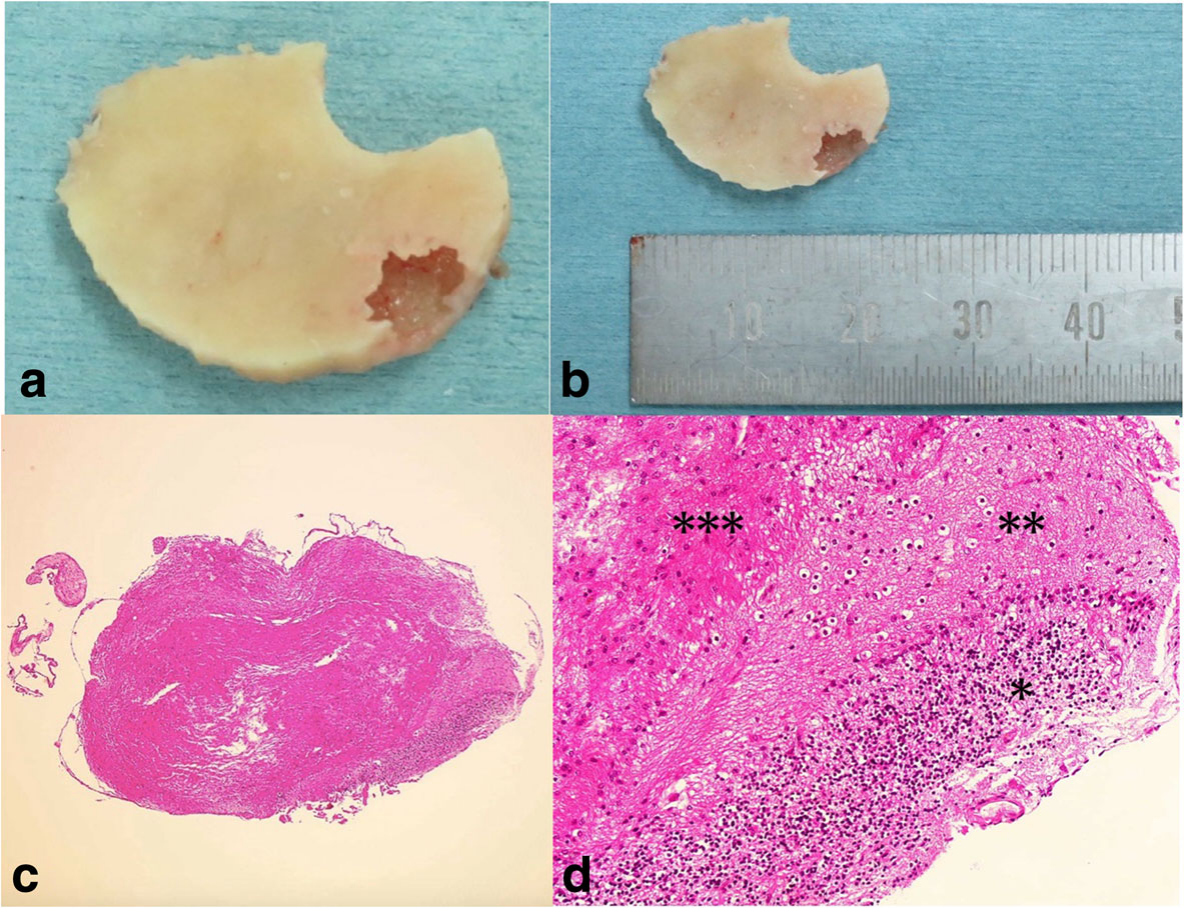
A photograph of the bone graft after craniotomy on the dural side (**a**, **b**) and findings of light microscopy (**c**, **d**). Low-power photomicrograph demonstrating the cerebellar cortex, which shows both the molecular layer and the granular layer (**c**). Meninges are evident around the tissue. Hematoxylin and eosin, original magnification × 40. High-power photomicrograph showing the granular layer (*), molecular layer (**), and white matter (***). Hematoxylin and eosin, original magnification × 200 (**d**)

Our patient underwent a right suboccipital craniotomy to create an opening 3 cm in diameter. After craniotomy, there were several small dural openings, causing some CSF leakage. The outer table of the detached bone was normal while the inner table was partially lacking, creating a tiny cavity filled with fragile reddish tissue (Fig. 2). The tissue which was obviously separated from the cerebellar tissue was resected to be a subject for pathological examination.

After successful microvascular decompression, the wound was closed using the bone graft for cranioplasty. Her postoperative course was uneventful and she was discharged without facial spasms or any neurological complication.

### Histopathological examination

The specimen exhibited a fragment of cerebellar tissue with gliosis, as well as necrotic bone and fibrous interstitial tissue. The cerebellar tissue consisted of cerebellar cortex and white matter. The cerebellar cortex clearly exhibited the molecular layer and the granular layer; however, Purkinje cells were depleted, and had been replaced by proliferation of Bergmann glia. The white matter was gliotic, and meninges were evident at the surface of the cerebellum. There were no features indicative of neoplasia.

## Discussion

Although some previous reports have referred to cerebellar ectopia, the definition itself has not been consistent. Caudal displacement of the tonsils due to Chiari malformation has been described as a manifestation of cerebellar ectopia in some previous cases [1, 2], including an extreme case of Chiari II malformation with complete caudal displacement of the cerebellum [1]. However, these cases should be referred to as displacement of the cerebellum, and not ectopia [3]. The term “heterotopic cerebellum” or “cerebellar heterotopia” has sometimes been used to indicate ectopic cerebellum. The terms *ectopic* and *heterotopic* are not synonymous but have precise definitions; ectopic cells occur *outside* their organ of origin whereas heterotopic cells are in an aberrant location *within* their organ of origin [4]. Ectopic cerebellum should be appropriately distinguished from heterotopia, which would include the more frequent focal entity, cerebellar cortical dysplasia.

Ectopic cerebellar tissue is very rare, and only 13 cases have been reported previously to the best of our knowledge (Table 1). Four of these cases were associated with neural tube defects [5–8] and two cases involved nasopharyngeal teratoma with ectopic cerebellar tissue in the suprasellar region [9, 10]. Six cases were unassociated with any other diseases or malformations. The locations of the ectopic cerebellar tissue included the fourth ventricle, orbit, frontal fossa, posterior fossa, suprasellar region, and sphenoid ridge [11–17]. Four of them presented with a mass effect, and one with epilepsy. All of the patients except one were children.

**Table 1:** Literature review of previous cases of cerebellar ectopia

Authors and year	Age, sex	Location	Symptoms or findings	Accompanied diseases or clinical manifestation	Therapy
Billings and Danziger, 1973 [11]	9 mos, M	4th ventricle	Enlargement of head	-	Resection
Marubayashi & Matsukado, 1978 [13]	27 d, M	Sphenoid ridge	Enlargement of head	Ectopia of cerebral and brainstem tissue	Resection
Suneson & Kalimo, 1979 [8]	21 mos, M	Cervicothoracic spinal cord	Swelling in the cervicothoracic region of the back	Myelocystocele and spina bifida	Resection and repair
Call & Baylis, 1980 [5]	At birth, F	Orbit	Proptosis	Suspected orbital meningoencephalocele	Resection
Sarnat *et al*., 1982 [7]	At birth, F	In frontal encephalocele	Forehead swelling	Multiple malformations of the CNS	(Autopsy)
Kagotani *et al*., 1996 [17]	2 mos, F	Orbit	Difficulty in right eyelid opening	Asymptomatic Chiari I malformation	Partial resection
Chung *et al*., 1998 [6]	14 mos, M	Cervical spinal cord	Scoliosis	Suspected split-cord malformation	Resection
Takhtani *et al*., 2000 [10]	At birth, F	Suprasellar	Optic neuropathy	Nasopharyngeal teratoma, choanal atresia, interhemispheric arachnoid cyst	Partial resection
Chang *et al*., 2001 [9]	4 mos, F	Suprasellar	Optic neuropathy	Nasopharyngeal teratoma	Resection
Okazaki *et al*., 2004 [16]	4 yrs, F	Suprasellar	Asymptomatic	-	Resection
Matyja *et al*., 2007 [14]	25 yrs, F	Anterior cranial fossa	Epilepsy	Hypertelorism, skull deformation	Resection
Nagaraj *et al*., 2012 [15]	5 yrs, M	Posterior cranial fossa	Headache	-	Resection
Gunbey *et al*., 2016 [12]	6 d, M	Posterior cranial fossa	Detected by screening, ultrasound imaging	Large posterior fossa cyst	-
Present case	46 yrs, F	Occipital bone	Asymptomatic	-	Resection

*CNS* central nervous system, *d* days, *F* female, *M* male, *mos* months, *yrs* years

Although the pathogenesis of ectopic glioneuronal masses has not been well understood, two main hypotheses have been classically proposed [16, 18, 19]. One of them is brain herniation or protrusion; that is, herniation of mature tissue from the neuraxis through a pre-existing pial defect [20]. The other is aberrant migration, that is, embryonic neuroepithelial tissue that aberrantly migrates into the subarachnoid space and subsequently develops into mature brain tissue.

It is noteworthy that primary diffuse leptomeningeal gliomatosis can arise from ectopic glial tissue, although this is very rare and often associated with progressive deterioration of clinical status [21–23]. A rare case of malignant glioma suspected to have arisen from ectopic glial tissue at the cavernous sinus has also been reported [24]. The possibility of malignant change of ectopic glial tissue is still worthy of consideration, even though intraosseous glioma has not been reported.

An intraosseous osteolytic lesion of the skull, such as that in the presented case, also needs to be distinguished from intraosseous neoplasms, such as meningioma, hemangioma, Langerhans cell histiocytosis, dermoid cyst, multiple myeloma, and malignant lymphoma. Images of such entities should be evaluated carefully focusing on the site, shape, and multiplicity of the lesion, the presence of accompanying soft tissue components, or any extracranial lesions [25].

A recent study presented a case of radiologically proven ectopic cerebellar tissue using diffusion tensor tractography and MR spectroscopy in a newborn [12]. If the tissue is big enough to be assessed by such neuroimaging tools and has connection with cerebellum, it is reasonable to diagnose ectopia with only radiological findings and without histopathological examinations.

Our patient had a tiny area of tissue within the occipital bone in the posterior cranial fossa, which was revealed histopathologically to possess typical cerebellar structure with gliosis. Preoperative radiological evaluation and our intraoperative findings demonstrated that this ectopic mass was surrounded by a normal subarachnoid space. Our observations and histopathological findings suggest that this ectopic cerebellar tissue could have protruded into the occipital bone through a dural defect and detached from cerebellum at an early stage of development, and then degenerated slightly or failed to undergo normal maturation, resulting in loss of Purkinje cells.

## Conclusions

We have described the first reported case of intraosseous ectopic cerebellar tissue in an adult woman with facial spasm but without any other neurological disorders. The clinical significance of these lesions should be clarified in future with further accumulation of cases.

## Abbreviations

CSF: Cerebrospinal fluid
CT: Computed tomography
MR: Magnetic resonance
